# On the interplay of curiosity, confidence, and importance in knowing information

**DOI:** 10.1007/s00426-023-01841-9

**Published:** 2023-06-06

**Authors:** Markus Wolfgang Hermann Spitzer, Janina Janz, Maohua Nie, Andrea Kiesel

**Affiliations:** 1 Department of Psychology, Martin-Luther University Halle-Wittenberg, Halle, Germany; 2 Albert-Ludwigs-University of Freiburg, Freiburg, Germany; 3 Department of Psychology, University of Basel, Basel, Switzerland

## Abstract

Curiosity appears to be the driving force for humans to find new information, but despite its general relevance, only a few studies investigated the underlying mechanisms of curiosity. Kang et al. (Psychol Sci 20(8):963–973, 2009) and Dubey and Griffiths (Psychol Rev 127(3):455–476, 2020) reported a relation between curiosity and confidence such that curiosity follows an inverted U-shaped function of confidence, with the highest curiosity on moderate confidence levels of knowing information. Given that replications of findings on curiosity are rare, this study sought to replicate these previous findings in two experiments, with the same stimulus material (Experiment 1) and new stimulus material using COVID-19-related information (Experiment 2). Based on theoretical predictions by Dubey and Griffiths (2020), we extended previous findings assessing the effect of the importance of information for the participant on the relationship between curiosity and confidence. Our findings replicated previous results in both experiments with the highest curiosity regarding information about which participants were moderately confident in knowing. Our extended analyses suggest that if information can be considered as important, then people are most curious about information when having very low-to-moderate confidence in knowing this information. However, if information is rated as rather not important, then curiosity is highest for information with moderate confidence in knowing the information. Together, these results emphasize the modulatory effect of perceived importance on the interplay between curiosity and confidence in knowing information.

**Supplementary Information:**

The online version contains supplementary material available at 10.1007/s00426-023-01841-9.

## Introduction

Curiosity^1^ affects our information-seeking behavior throughout the day (e.g., curiosity determines which person our eyes fixate on at the bus stop or which link we click on when browsing the web), shapes the long-term progress of scientific discovery, and has been described as the essence of science. Given the persistent influence of curiosity throughout our daily lives (Berlyne, 1950; Kang et al., 2009; Loewenstein, 1994), and given that scientists have been curious about curiosity for a long time (e.g., Hall & Smith, 1903), it appears perplexing that only recently, studies have begun to systematically investigate the underlying mechanisms of curiosity (Dubey & Griffiths, 2020; Gottlieb & Oudeyer, 2018; Gottlieb et al., 2013; Kang et al., 2009; Kidd & Hayden, 2015; Wojtowicz & Loewenstein, 2020).

In this study, we first replicated the core findings of the study by Kang et al. (2009), with the same stimulus material, which investigated curiosity as a function of confidence. We then extended these findings from Kang et al. (2009) by additionally investigating the role of the importance of information on the relationship between curiosity and confidence. Finally, we replicated these results with a second experiment and different stimulus material. The content of the new stimulus material was about the ongoing COVID-19 pandemic, because we wanted to use material about which almost everybody is highly concerned.

### Curiosity as a function of confidence

The information-gap theory (Loewenstein, 1994) sees the roots of curiosity in the gap between already-known information about a topic and the knowledge level one aspires to. Loewenstein proposed that curiosity is like the feeling of hunger, but for knowledge: a small “bite of knowledge” increases the hunger for more information, but after gaining more and more information, the hunger is satiated, and thus, curiosity decreases. Inspired by this theory, Kang et al. (2009) examined curiosity as a function of confidence in knowing information. They hypothesized that if people are moderately confident in knowing information, they will be most curious about it. In contrast, if they know nothing, then they cannot be curious. And if they feel to know all about something, they will feel not curious about it anymore. To investigate this hypothesis, Kang et al. (2009) used a set of 40 trivia questions regarding knowledge of the information to examine the information-gap theory.^2^ The questions were shown to cover different curiosity levels during pretesting. Specifically, their experiments followed the same basic procedure for all 40 questions: (1) participants were presented with a trivia question and were instructed to guess the corresponding answer in their head; (2) participants rated their curiosity on a scale from 1 to 7; and (3) participants rated their confidence concerning their guessed answer on a scale from 0 to 100%. The results of their first experiment mimicked the hunger metaphor of the information-gap theory for curiosity, with an inverted U-shaped relationship between curiosity and confidence ratings, with little curiosity at low and high confidence ratings and maximum curiosity ratings at medium confidence levels.

In addition to these self-rating measurements, Kang et al. (2009) hypothesized that situations in which curiosity about knowing something is high, should be associated with a rewarding effect for the learner, as more—presumably important—information is gained during these phases. Thus, they hypothesized that curiosity would be associated with a higher probability to spend resources to learn new information to close information gaps. They tested this hypothesis with another experiment, which was similar to the first one but during which participants could spend time (or tokens) for information and thereby indicating their curiosity through their behavior. In this experiment, the first phase of the experiment was the same as in the first experiment. After the presentation of the 40 trivia questions, each question was presented again and participants typed in their initial guesses. Then, they chose whether they wanted to see the answer. One set of participants had the opportunity to wait for revealing the answer (time condition) and another set of participants had the opportunity to pay for revealing the answer with tokens (token condition). If participants were unwilling to wait or pay for the answer, they were able to choose to continue directly with the next question without seeing the right answer. With this setup, the authors sought to investigate whether participants would trade off gaining information against the cost of staying longer in the experiment or spending tokens. In line with their hypothesis, results revealed that participants were more willing to spend time or tokens with increasing curiosity.

In sum, Kang et al. (2009) provided evidence for the link between curiosity and confidence as an inverted U-shaped function following the information-gap theory by Loewenstein (1994). They also showed that the self-ratings on curiosity were indeed a valid measure, as participants were more willing to spend resources to learn information when being curious about it. However, the information-gap theory is also limited by the idea that individuals or animals can only be curious about the information that is already partly known. In terms of the hunger metaphor, a first bite is needed to elicit an appetite for knowledge. Thus, if no prior knowledge exists, individuals or animals will not be curious. Yet, being curious about something completely new has been addressed by other theories described in the following section.

### Curiosity as a function of novelty

Besides the information-gap theory from Loewenstein (1994), other observations suggest that the novelty of information has a vital effect on curiosity and that people gain intrinsic rewards and satisfaction when they learn about something new. In contrast to the information-gap theory, the novelty theory suggests that curiosity is highest for novel information. Evidence on this account comes from early studies on curiosity from Berlyne (1950, 1966). Berlyne found that animals, but also humans, are more curious about new stimuli (Berlyne, 1950, 1966). Similar observations were made by Smock and Holt (1962) who showed that toddlers play longer with new toys compared to already-known toys (Smock & Holt, 1962). One issue of such novelty-based accounts is that they must assume that learning about novel stimuli is always beneficial, which is not necessarily the case (Gottlieb et al., 2013). Furthermore, novelty theories cannot explain why people have familiarity preferences or sometimes even avoid novel information which may be useful to them (Golman et al., 2017; Kidd & Hayden, 2015; Loewenstein, 1994). For example, many people, who are already in medical care, annually omit the opportunity to be tested for HIV, even if tests are for free and not associated with any additional effort (Sweeny et al., 2010; Tao et al., 1999).

### A rational model combining the two perspectives of complexity and novelty

While evidence exists in favor of the information-gap theory and in favor of the novelty theory, Dubey and Griffiths (2020) recently argued that these two dominant theories are not mutually exclusive and combined both theories within one theoretical computational framework. They suggest that a key factor that determines the level of curiosity is the value of knowing information. This value, however, depends on the probability that this information will (or will not) occur again in the future. If the information will occur again, it is valuable knowing it. Otherwise, it is not valuable to know it. Dubey and Griffiths (2020) tested this prediction, using a similar paradigm and the same stimulus material as in the study by Kang et al. (2009) in an online study that had three phases. Phase 1 was based on the core procedure from Kang et al. (2009)—participants were shown a question, rated their curiosity (0–7), and their confidence in knowing the right answer to that question (1–100%) for all 40 questions in a row. In Phase 2 each question was presented again, and participants could choose to reveal the correct answer in exchange for waiting 10 s (with the decision to wait as an indicator of curiosity) or otherwise continue directly with the next question.^3^ In Phase 3, 10 out of the 40 questions were shown again and the participants had to type in the respective answer/their guess (they received a small reward per right answer within a set time frame to prevent online research on this question).

To test their hypotheses about whether the function of curiosity and confidence further interacts with whether the information will, or will not, occur again in the close future, and thus is more or less useful to maximize rewards, participants were assigned to two conditions. These two conditions differed in how the 10 questions were sampled in Phase 3. In the uniform condition, each question was equally likely to occur in Phase 3. In the confidence condition, however, the probability of the 10 questions occurring in Phase 3 depended on participants' confidence ratings in Phase 1, with a higher confidence rating leading to a higher probability of occurrence in Phase 3. Importantly, participants were told about each respective sampling process before Phase 2. As participants were explicitly instructed before Phase 2 about the sampling procedure in Phase 3, Dubey and Griffiths (2020) hypothesized that participants in the confidence condition should be more curious about information in Phase 2 they were moderately confident in knowing the answer, reflected with an inverted U-shaped function between curiosity and confidence (as these questions were more likely to occur again in Phase 3, where they will be able to gain rewards for a correct answer). In addition, participants in the confidence condition would be less curious about the information they were either confident in knowing, as they already knew the answer to these questions, or not confident at all in knowing the answer, as these questions were unlikely to occur again in Phase 3, and thus were expected with fewer rewards. However, participants from the uniform condition (where information was equally likely to occur again in Phase 3) were supposed to be most curious about unknown information (low confidence ratings in Phase 1), reflected in a negative relationship between curiosity and confidence, as learning the answer about these questions would help to maximize rewards in the close future (i.e., in Phase 3).

The results were in line with the predictions from their rational computational model. Results from Phase 1 first showed that curiosity followed an inverted U-shaped function for both groups of participants (as in Experiment 1 from Kang et al., 2009). Results for Phase 2 differed between groups: participants from the confidence group were most curious about information when their confidence was moderate in knowing the answer. Participants from the uniform group were most curious about information when their confidence was low in knowing the answer.

Together, these results support the prediction that curiosity about information depends on the value of knowing this information. This critical aspect allows for explaining the findings from Dubey and Griffiths (2020) in the view of perceived importance of information. Specifically, participants from the confidence condition were explicitly told that information they were more confident about was more important in the close future (because of the higher likelihood to appear in Phase 3 in which knowing the correct answer/information would be rewarded). This indicated that information on moderate confidence levels was important to maximize rewards, as this information was likely to occur again in Phase 3. On the other hand, participants from the uniform condition were told that information from all confidence levels was equally important to maximize rewards in Phase 3. Thus, information with the lowest confidence levels was most important, as the largest information gaps existed here, and closing these gaps would lead to maximal rewards.^4^

In sum, these results suggest that people should be most curious about information gaps, which are perceived to be important. In other words, irrespective of whether confidence in information is low or moderate, as long as these information gaps are perceived as important, they should induce curiosity. Critically, the results from Dubey and Griffiths (2020) indicate that people can, and do, estimate the value of knowing a particular piece of information, even if their confidence in knowing the answer is low. This implies that people have—at least in some situations—a metacognitive estimation of the importance of knowing information, even if they hardly know anything about this information other than that it is important.^5^ This further implies that asking people on whether knowing information would be important or not, in addition to asking them about their confidence in knowing the answer, may be a critical predictor for curiosity about knowing this information and consequently, their willingness to close this information gap. Here, we sought to explicitly test the influence of importance on the relationship between curiosity and confidence.

### The present research

This study comprised two experiments. For each experiment, we conducted a two-step analysis approach to (1) replicate the findings by Kang et al. (2009) and (2) extend these findings by investigating whether the importance of knowing information interplays with the relationship between curiosity and confidence. In the following, we first describe the analyses conducted for Experiment 1 followed by a description of analyses for Experiment 2.

In the first step of analyses for Experiment 1, we sought to replicate the main behavioral findings by Kang et al. (2009). For this replication, the same stimulus material as in Kang et al. (2009), was applied (Experiment 1). Specifically, in the first phase of Experiment 1, participants were asked trivia questions and subsequently rated these questions on a curiosity scale (1–7) and a confidence scale (0–100%). In the second phase, participants were asked if they would like to wait for 5–25 s to reveal the answer to the question or if they would like to just skip the answer to proceed with the experiment.

We carried out two analyses also reported by Kang et al. (2009). First, we investigated curiosity as a quadratic function of confidence and expected curiosity to follow an inverted U-shaped function of confidence, reaching its maximum when confidence is moderate (see Fig. 1C in Kang et al., 2009). Second, we investigated participants’ probability to reveal an answer as a function of curiosity^6^ (see Fig. 5 in Kang et al., 2009), and expected more willingness to spend effort (in terms of time) to reveal an answer on high curiosity levels^7^ (see Fig. 5 in Kang et al., 2009).

In the second step of analyses, we sought to extend these findings by additionally investigating the effect of the importance of knowing information on curiosity and participants’ probability to reveal an answer. Therefore, we directly asked participants to indicate the importance of knowing the answer to each question in this study. We thus added an importance scale (1–7) to the first phase of both experiments, respectively. Since the trivia questions elicited different levels of curiosity in the study by Kang et al. (2009), we reasoned that the perceived importance of information may also vary.

We then tested whether the addition of the importance variable fitted the data better, compared to the model which replicated results by Kang et al. (2009). We also explored the goodness of fit of several other models with curiosity as the dependent variable with each other (see Table 1). We predicted that the importance of knowing information significantly interplays with curiosity. Therefore, we expected that adding this variable should lead to an increased model fit, compared to the models reported in the first step of our analyses.

**Table 1:** Models predicting curiosity and associated BIC scores and AIC scores of each model and Experiment 1 (Exp. 1) and Experiment 2 (Exp. 2)

Model formula	Exp 1: BIC	Exp 1: AIC	Exp 2: BIC	Exp 2: AIC
${\text{Curiosity}}\, = \,b_{1} *{\text{confidence}}^{{2}} \, + \,b_{2} *{\text{confidence}}\, + \,b_{3} *{\text{importance}}$	**2021.8**	1984.1	**2047.2**	2010.1
${\text{Curiosity}}\, = \,b_{1} *{\text{importance}}$	2081.4	2062.5	2058.6	2040.1
${\text{Curiosity}}\, = \,b_{1} *{\text{confidence}}^{{2}} \, + \,b_{2} *{\text{confidence}}$	2251.1	2227.5	2139.6	2116.4
${\text{Curiosity}}\, = \,b_{1} *{\text{ confidence}}$	2311.3	2292.4	2151.4	2132.9
${\text{Curiosity}}\, = \,b_{1} *{1}$	2310.2	2296.1	2142.4	2128.5

BIC scores of Experiment 1 (Exp. 1) are ranked in ascending order (from lowest to highest). Lower BIC scores indicate better model fits. The best-fitting models with respect to BIC are marked in bold

**Fig. 1:**
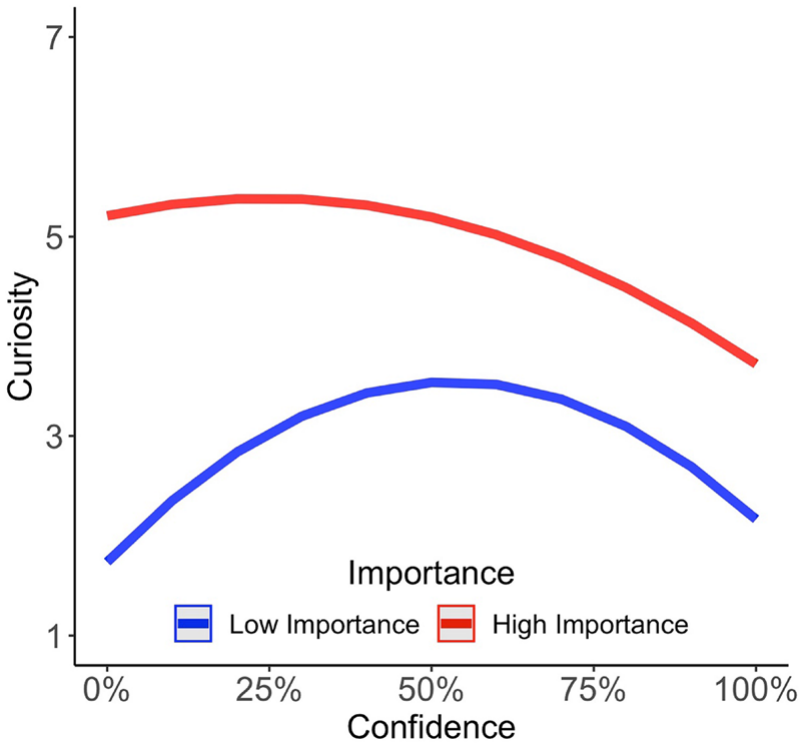
Hypothetical plot of curiosity predicted by confidence and importance. Based on Dubey and Griffiths (2020), these hypothetical predictions aim to integrate the information-gap theory and the novelty theory within one framework by assessing the modulatory effect of the importance of knowing about information. If information is rated as important (red line) curiosity is predicted highest for low-to-moderate confidence in knowing information, in line with the novelty theory. If information is rated as less important (blue line) curiosity overall drops and is predicted highest for moderate confidence in knowing information, in line with the information-gap theory

In particular, the rational model of Dubey and Griffiths (2020) theoretically predicts that both curiosity theories can be integrated within one model when assessing the value of information. Here, we assessed the value of information by asking participants about the subjective importance of knowing information. We thus predicted that for information perceived as important, curiosity should be highest for low-to-moderate confidence in knowing this information (see red curve in Fig. 1), as closing these information gaps may be associated with high future rewards. However, with decreasing importance of knowing information, we expected this curve to drop down and shift toward an inverted U-shaped function of curiosity and confidence (see blue curve in Fig. 1), as low confidence in knowing information and perceiving this information as not important may actually indicate that people know nothing about this information at all and are thus not curious about it. But if people would already know a bit of information, they may be hungry about knowing all of it—as suggested by the information-gap theory—even if the information would be perceived as less important.

In another analysis, we explored the interplay of (1) the importance of knowing information, (2) curiosity, and (3) confidence in participants’ probability to reveal an answer (see Table 2). Therefore, we carried out several plausible combinations of models to explore whether the addition of an importance term increased the model fit to predict participants’ probability to reveal an answer. Note that we assessed all three independent variables as the analyses reported by Kang et al. (2009) and Dubey and Griffiths (2020) differed with respect to the regressor variable when predicting participants’ probability to reveal an answer. Kang et al. predicted the probability to reveal an answer with curiosity (see Fig. 5 in Kang et al., 2009), while Dubey and Griffiths predicted the probability to reveal an answer with confidence for two different groups (see Fig. 6B in Dubey & Griffiths, 2020). We report the results of the model with the best fit.

**Table 2:** Models predicting the decision to reveal an answer and associated BIC scores of each model

Model formula	Exp1: BIC	Exp1: AIC	Exp 2: BIC	Exp 2: AIC
${\text{Decision}}\, = \,b_{1} *{\text{curiosity}}\, + \,b_{2} *{\text{importance}}$	**936.4**	912.8	930.0	906.9
${\text{Decision}}\, = \,b_{1} *{\text{curiosity}}$	937.2	923.1	917.6	903.7
${\text{Decision}}\, = \,b_{1} *{\text{curiosity}}\, + \,b_{2} *{\text{confidence}}^{{2}} \, + \,b_{3} *{\text{confidence}}$	951.5	918.5	918.5	876.0
${\text{Decision}}\, = \,b_{1} *{\text{curiosity}}\, + \,b_{2} *{\text{confidence}}^{{2}} \, + \,b_{3} *{\text{confidence}}\, + \,b_{4} *{\text{importance}}$	967.1	905.8	929.4	869.2
${\text{Decision}}\, = \,b_{1} *{\text{confidence}}^{{2}} \, + \,b_{2} *{\text{confidence}}\, + \,b_{3} *{\text{importance}}$	984.4	951.5	**906.8**	874.4
${\text{Decision}}\, = \,b_{1} *{\text{importance}}$	996.8	982.6	955.3	930.4
${\text{Decision}}\, = \,b_{1} *{\text{confidence}}^{{2}} \, + \,b_{2} *{\text{confidence}}$	1067.5	1048.7	923.7	905.2

Lower BIC scores indicate better model fits. BIC scores of Experiment 1 (Exp. 1) are ranked in ascending order (from lowest to highest). The best fitting models with respect to BIC are marked in bold

Experiment 2 considered new stimulus material to test whether the results obtained in Experiment 1 generalize to other stimulus material. Therefore, we exposed participants to a set of COVID-19-related questions (Experiment 2). Except for this change in stimulus material, the setup of Experiment 2 was the same as in Experiment 1. The analyses followed the same procedure as for Experiment 1. We first sought to replicate the findings by Kang et al. (2009) and then sought to extend these findings by considering the importance of knowing information in two model comparison analyses (see Tables 1, 2). We describe the results of the winning model.

## Experiment 1

### Method

#### Participants

We recruited 43 German participants (15 women, 28 men, *M*_age_ = 28.13 years; range 18–43) via Prolific to conduct the online study. All participants provided informed consent prior to the onset of the study. When planning the experiment, we reasoned that doubling the sample size used in Experiment 1 by Kang et al. (2009) should be sufficient to reveal the assumed inverted U-shaped relationship between curiosity and confidence. After we collected the data, we conducted a post hoc power analysis with the simr package in R (Green & Macleod, 2016). We describe the results of this power analysis in the results section.

#### Stimuli

The stimuli used in Experiment 1 were the same trivia questions as used in the studies of Kang et al. (2009) and Dubey and Griffiths (2020). One answer was changed to be up to date (Question: Which country has the highest percentage of women in the government? Original answer Belgium was changed to Ruanda). These questions were designed to elicit curiosity (for more details on these questions, see Kang et al., 2009). Another example of a trivia question is: “What instrument was invented to sound like a human singing?”, Answer: “Violin”. Compared to the previous two studies and following the advice from one of the authors from the previous studies, only 20 trivia questions were used in this experiment, as 40 trivia questions may be too many to keep participants’ curiosity high throughout the whole experiment and thus, we intended to prevent participants from attention drift on later questions. These 20 questions are listed in the supplementary material.

#### Procedure

**Fig. 2:**
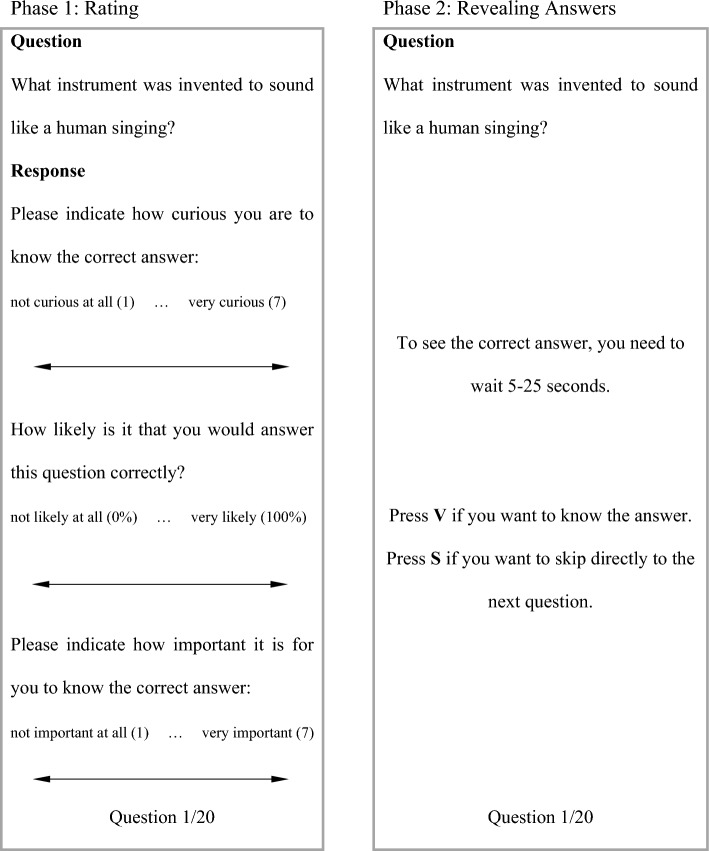
First phase (rating) and second phase (revealing answer) of Experiment 1

The experiment consisted of two phases, a “rating phase” and a “revealing answer” phase. Stimuli examples of each of these two phases are depicted in Fig. 2. Participants became familiar with the basic design of the whole experiment with a practice question at the start of the experiment which was always the same (“What animal can shed up to 30,000 teeth in its lifetime?”, Answer: Shark). They were asked to guess the answer to this question in their mind and self-report their curiosity level (1–7), confidence about knowing the answer (0–100%), and the importance of knowing the answer to this question (1–7). After that, participants were presented with 20 trivia questions one after another. The order of the 20 questions was randomized for each participant.

After this first rating phase, the second phase started. Each question was presented again one after another and participants were told that they could either wait for a period of time (between 5 and 25 s) to learn the answer or they could choose to skip directly to the next question, but then they would not see the correct answer to the question (see Fig. 2). Please note that the waiting time would vary randomly, as in Kang et al. (2009), for each trial and could be any amount of time from 5 to 25 s. Participants were told that any amount of waiting time would be equally likely. Experiment 1 took approximately 15 min to complete.

#### Data analysis

We conducted the statistical analysis in the R environment for statistical computing (R Core Team, 2013; RStudio Team, 2015). All linear regression analyses were performed using the lmerTest (Kuznetsova et al., 2017) package. Logistic regression analyses were fitted with the lme4 (Bates et al., 2014) package. Plots were generated using the sjPlot package (Lüdecke, 2020).

The first analysis addressed the observation by Kang et al. (2009) and Dubey and Griffiths (2020) of an inverted U-shaped function between individually normalized curiosity and confidence. This analysis followed the identical procedure as ofKang et al. (2009).^8^ First, the raw curiosity ratings were normalized for each participant using the following equation:

1 $$ {\text{normalized curiosity }} = \, \left( {{\text{raw curiosity value }}{-}{\text{ curiosity mean}}} \right)/ \, \left( {\text{curiosity standard deviation}} \right). $$

Confidence ratings were re-scaled to range from 0 to 1. Then, a hierarchical regression model was computed with the re-scaled confidence factor as an independent variable with a 1st and 2nd order polynomial term. In detail, the regression equation for fixed effects used in this model was:

2 $$ {\text{curiosity }} = b_{1} *{\text{confidence}}^{{2}} + b_{2} *{\text{ confidence}}{.} $$

Additionally, a random intercept was added for each participant to account for the overall variance in curiosity between participants. No random slope term was added to the model, as this more complex model, with a random slope for confidence, did not account for more variance. We expected to replicate the results of Kang et al. (2009) of an inverted U-shaped relationship between confidence and curiosity, reflected with a significant and negative quadratic coefficient for confidence.

Based on the motivation to replicate results reported in Experiment 3 by Kang et al. (2009), a second analysis investigated the effect of curiosity on the decision to reveal the answer or not. A logistic regression was fitted to the data, with the normalized curiosity factor as the independent variable and the decision (see answer vs. skip to next question) as the dependent variable. The logistic regression model was:

3 $$ {\text{decision }} = b_{1} *{\text{curiosity}}{.} $$

As in Analysis 1a, a random intercept term was added for each participant.

After this first step of analyses, which aimed to replicate results by Kang et al. (2009), we sought to extend these findings, asking whether participants’ importance ratings on trivia questions influenced their curiosity ratings and their subsequent willingness to spend resources (time) to learn the answer to a specific question. In particular, we extended the hierarchical linear regression model described in Eq. (2) with an additional importance variable as a main and interaction effect:

4 $$ {\text{curiosity }} = b_{1} {\text{confidence}}^{{2}} + b_{2} {\text{confidence }} + b_{3} {\text{importance }} + b_{4} {\text{confidence}}^{{2}} *{\text{ importance }} + b_{5} {\text{confidence }}*{\text{ importance }} + b_{6} . $$

As this model was an extension of the model described in Eq. (2), we compared the goodness of fit of this model concerning the Bayesian Information Criterion (BIC), with smaller BICs reflecting better model fits (Burnham & Anderson, 2002), to investigate whether the addition of the importance in knowing variable increased the model fit. BIC differences larger than 10 indicate that the model with a lower BIC score fits the data better. We computed further indicators for model fits such as the Akaike information criterion (AIC) if models revealed similar BIC scores (BIC differences below 10) and reported the model with the lowest AIC if the BIC of models differed by less than 10. Note that smaller AIC scores indicate better model fits. We assessed the BIC metric as the first instance of model comparison as BIC penalizes more complex models compared to AIC.

We additionally compared the fits of these two models with curiosity as the dependent variable with two further models which varied concerning the independent variables: an intercept-only model and an importance-only model (see Table 1 for model description and BIC scores). As in the previous models, a random intercept for participants was added as a random effect. No random slope terms were added as this did not improve the model fits concerning BIC.

We followed the same logic of model comparisons for extending the model described in Eq. (3) with the second dependent variable waiting time. Importantly, Kang et al. (2009) investigated waiting time as a function of curiosity, while Dubey and Griffiths (2020) investigated waiting time as a function of confidence and group. We thus considered both of these independent variables as well as the importance variable and compared all combinations of models with respect to BIC and reported the model with the best fit (see Table 2 for model description and BIC scores). Note that we also considered an intercept-only model. All models were fitted with a random intercept for participants and no random slope terms as the addition of random slopes did not improve model fits.

### Results

The same inclusion criteria were applied as in Dubey and Griffiths (2020). All participants who chose to reveal the answers to all 20 questions and those who never chose to reveal the answer to any questions were removed. Based on these criteria, one participant was removed. Another participant was additionally excluded as this participant provided the same answer to all questions. The final data set of Experiment 1 thus consisted of a total of 41 participants.

#### Analysis 1a: replication results: curiosity as a function of confidence

**Fig. 3:**
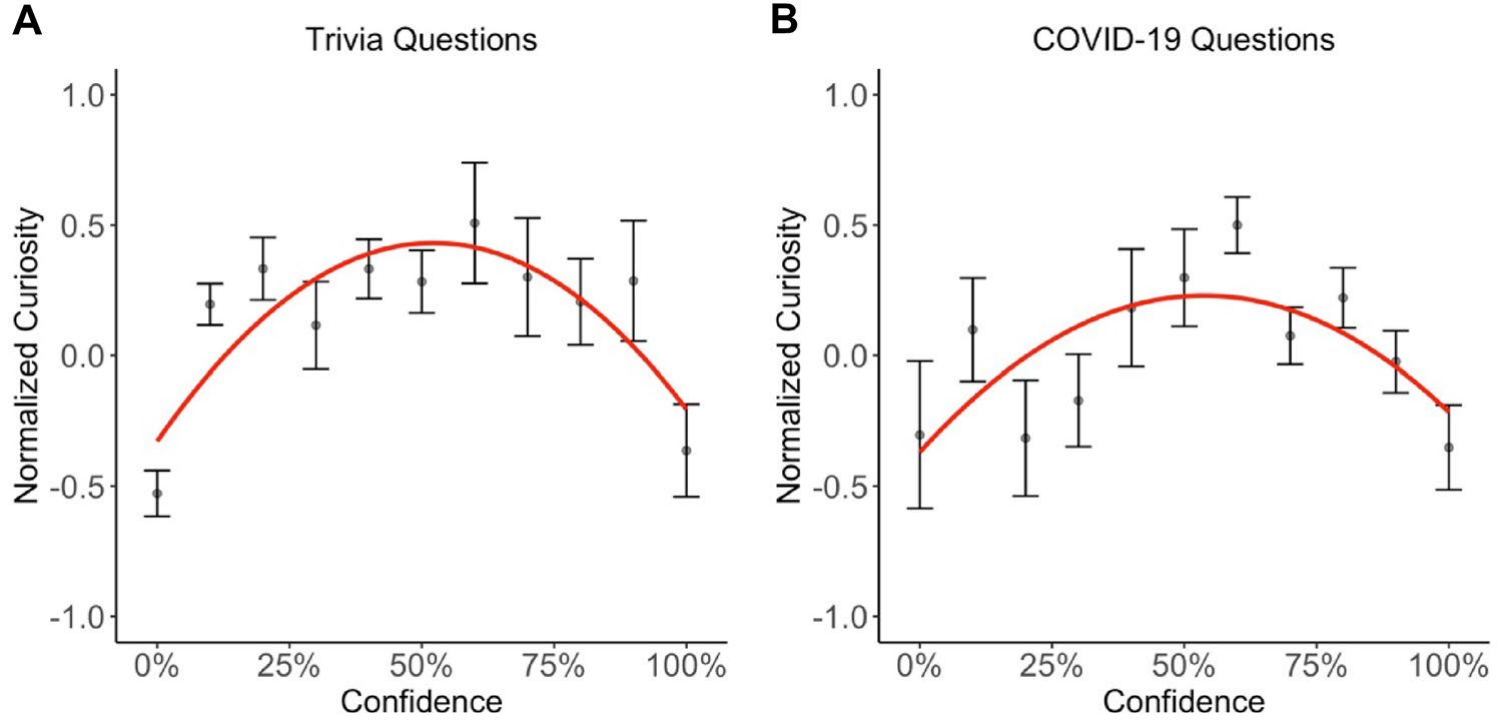
Curiosity as a function of confidence for trivia questions (**A**) and COVID-19 questions (**B**). Results for trivia questions replicated previous results by Kang et al. (2009). Results for COVID-19 questions extended these findings to another stimulus material. In both experiments, moderate confidence levels elicited the highest curiosity levels. The red line indicates the regression fit. Error bars indicate the standard error of the mean

The results of this analysis are reported in Table 3. Consistent with Kang et al. (2009), curiosity followed an inverted U-shaped function of confidence, peaking when confidence was approximately 0.50 (see Fig. 3), with a significant quadratic coefficient for confidence (*b*_1_ estimate = − 7.43; *t* = − 7.94; *p* < 0.001) and a significant coefficient for confidence (*b*_2_ estimate = 2.79;* t* = 2.98; *p* = 0.003).

**Table 3:** Curiosity on trivia questions predicted by confidence and importance

Predictors	Curiosity				Curiosity			
	*B*	SE	*t* value	*p*	*b*	SE	*t* value	*p*
Intercept	− 0.00	0.03	− 0.01	0.996	− 0.64	0.07	− 8.85	** < 0.001**
Confidence [1st degree]	2.79	0.94	2.98	**0.003**	4.03	1.37	2.94	**0.003**
Confidence [2nd degree]	− 7.43	0.94	− 7.94	** < 0.001**	− 7.73	1.43	− 5.40	** < 0.001**
Importance					0.34	0.02	18.13	** < 0.001**
Confidence [1st degree]: Importance					− 2.69	0.47	− 5.76	** < 0.001**
Confidence [2nd degree]: Importance					1.24	0.47	2.62	**0.009**
*N*	41 _participants_				41 _participants_			
AIC	2227.592				1984.182			

A smaller AIC score indicates a better model fit

Numbers are in bold for all p-values below 0.05

#### Analysis 1b: replication results: probability of revealing the answer as a function of curiosity

**Fig. 4:**
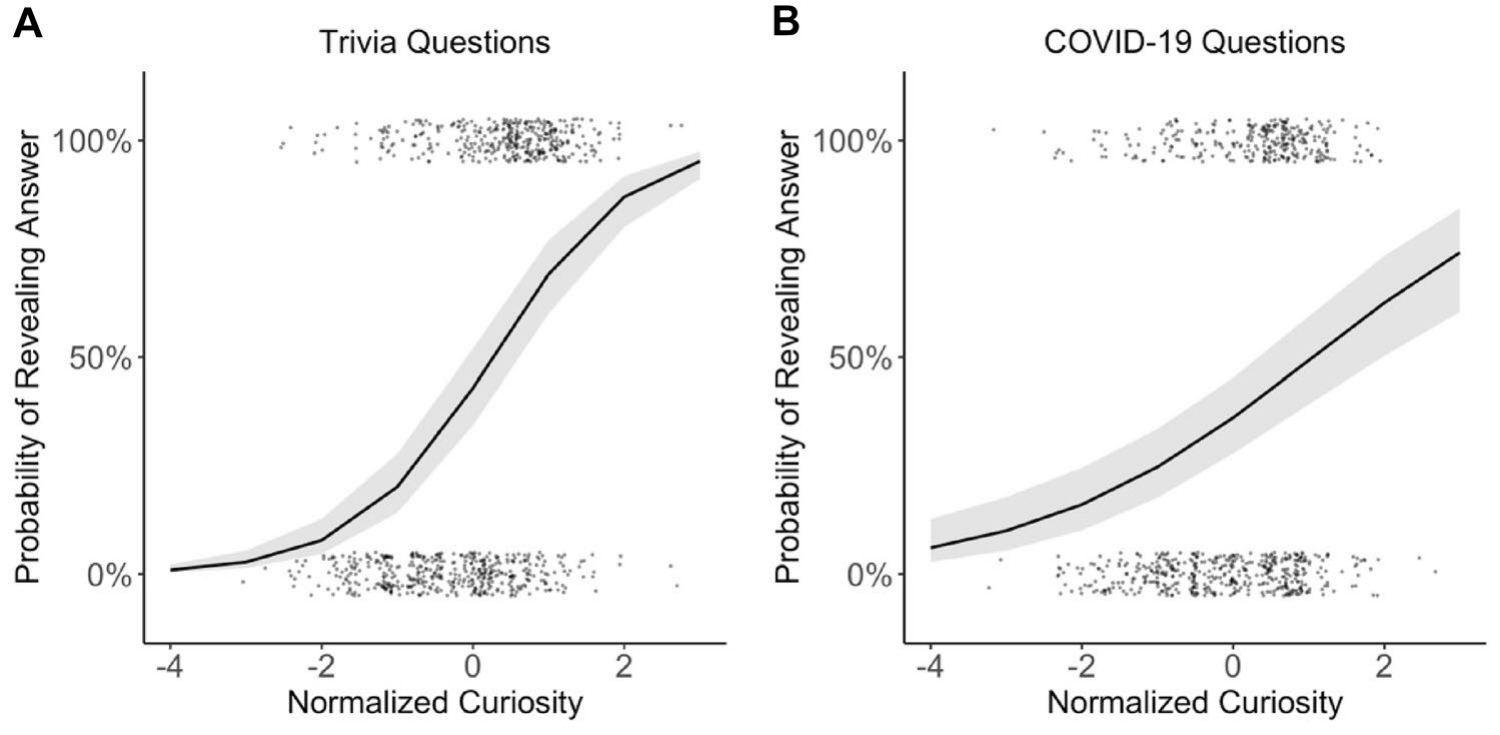
Probability of revealing the answer as a function of curiosity for trivia questions (**A**) and COVID-19 questions (**B**). In both experiments, participants were more likely to select to reveal the answer to questions with increasing curiosity. Black lines indicate the logistic regression line. The shaded area around the curves indicates the standard error of the mean. Points represent the data of individual participants jittered around 0% and 100% for presentation purposes

The results of this analysis are reported in Table 4. A logistic regression was then applied to analyze the effect of normalized curiosity on the decision of whether to reveal answers for trivia questions (see Fig. 4). In line with the results of Kang et al. (2009), results indicated a significant effect of normalized curiosity on the probability to reveal an answer (*b* = 1.09; *z* = 11.13; *p* < 0.001). The positive relationship between curiosity and the decision to reveal the answer was also indicated by a positive and significant correlation of *r* = 0.38, *p* = 0.015, and was in line with the reported correlation of *r* = 0.44 by Kang et al. (2009).

**Table 4:** The decision to reveal an answer (decision) on trivia questions predicted by curiosity and importance

Predictors	Decision				Decision			
	*b*	SE	*z* value	*p*	*b*	SE	*z* value	*p*
Intercept	− 0.28	0.19	− 1.53	0.126	− 0.79	0.23	− 3.46	**0.001**
Curiosity	1.09	0.10	11.13	** < 0.001**	0.83	0.15	5.75	** < 0.001**
Importance					0.23	0.07	3.46	**0.001**
Curiosity: importance					0.04	0.06	0.65	0.513
*N*	41 _participants_				41 _participants_			
AIC	923.107				912.857			

A smaller AIC score indicates a better model fit

Numbers are in bold for all p-values below 0.05

#### Model comparison results

Table 1 describes all models with curiosity as the dependent variable which were compared against each other concerning their BIC. Table 2 shows all models with the decision to wait for an answer as the dependent variable and the respective BIC scores. Curiosity was best explained by a model which included the 2nd order polynomial confidence variable and importance variable as well as the interaction between these two variables. Participants’ decision to wait for an answer was best predicted by a model of curiosity and importance.^9^ The results of these two winning models are described below as well as in Tables 3 and 4.

#### Analysis 1c: curiosity as a function of confidence and importance

**Fig. 5:**
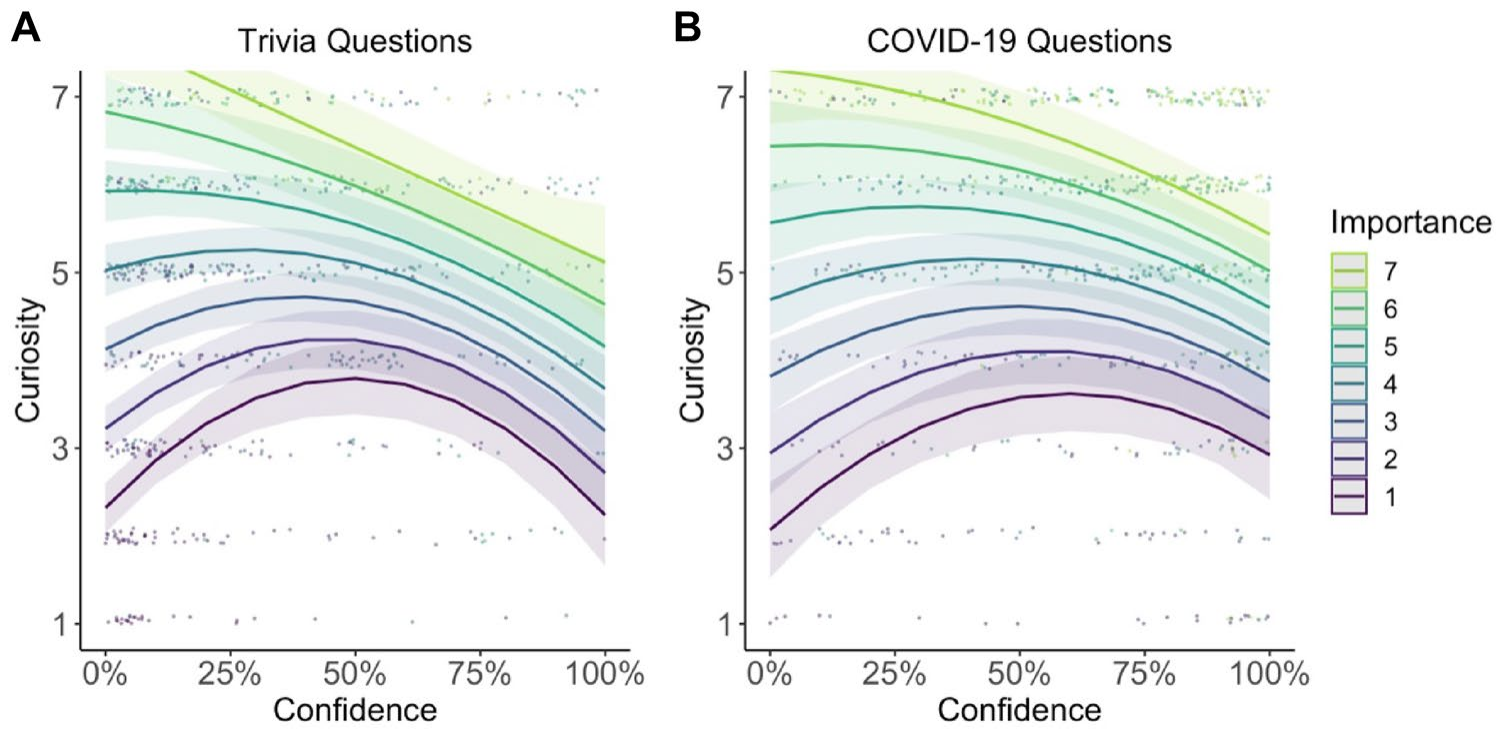
Curiosity as a function of confidence and importance for trivia questions (**A**) and COVID-19 questions (**B**). If participants rated the answer to the question as important, curiosity was highest for low-to-moderate confidence levels represented with a negative regression line. If participants rated the answer to the question as less important, curiosity followed an inverted U-shaped function of confidence. Solid lines indicate the logistic regression line. The shaded area around the curves indicates the standard error of the mean. Points represent the data of individual participants jittered for each curiosity level for presentation purposes. Also see Figure S1 for a similar visualization with a median split on importance (low and high)

Curiosity was best predicted by confidence in knowing information and the importance of this information (see Table 3 for the results). In particular, the analysis revealed a significant main effect of confidence on the quadratic term (*b*_1_ = − 7.73; *t* = − 5.40; *p* < 0.001), and the linear term (*b*_2_ = 4.03; *t* = 2.94; *p* = 0.003). In line with our predictions about the influence of importance on curiosity, the main effect of importance was significant (*b*_3_ = 0.34; *t* = 18.13; *p* < 0.001). In addition, the interaction between confidence and importance was significant, reflected by a significant quadratic term (*b*_4_ = 1.24; *t* = 2.62; *p* = 0.009) and a significant linear term (*b*_5_ = − 2.69; *t* = − 5.76; *p* = < 0.001). This interaction was in line with our predictions and showed that curiosity would asymptote on a high level for importance and low-to-moderate confidence ratings and decrease with higher confidence ratings. Furthermore, on lower importance ratings, curiosity followed an inverted U-shaped function of confidence (see Figs. 5, S1).

#### Analysis 1d: probability of revealing the answer as a function of curiosity and importance

Results of a logistic regression indicated a significant effect of normalized curiosity on the probability to reveal an answer (*b* = 0.83; *z* = 5.75; *p* < 0.001), with a positive relationship between curiosity and the decision to reveal the answer (also see Table 4). The main effect of importance on the willingness to spend time to reveal the answer was significant (*b*_3_ = 0.23; *z* = 3.46; *p* < 0.001), with a higher probability to reveal the answer with increased importance ratings. The interaction between curiosity and importance was not significant (*b*_4_ = 0.04; *z* = 0.65; *p* = 0.513).

#### Power analysis

We simulated the post hoc power of these results with the simr package in R (Green & Macleod, 2016) to inform further experiments on the robustness of the results reported in this experiment. These power simulations were conducted on the sample size of the final 41 participants. Please note that we used the normalized values and the same regression models as in the analysis described above for the power simulations. A first power analysis revealed that nine participants would be needed to reveal the observed effect of *b* = − 7.4 suggesting an inverted U-shaped relationship between curiosity and confidence observed in this study with a power of 80% for an alpha level of 0.05. We also simulated the power for curiosity as a function of importance and confidence. This simulation suggested a power of 85% for an interaction effect between importance and the quadratic confidence term of *b* = 1.24 for an alpha level of 0.05.

## Discussion

The results of the first two analyses of Experiment 1 replicated Kang et al.’s (2009) findings, showing that curiosity followed an inverted U-shaped function between curiosity and confidence^10^ (see Fig. 3) and participants were more likely to spend time to learn information which they were more curious about (see Fig. 4). While these findings supported the information-gap theory (Loewenstein, 1994), we tested whether participants’ perceived importance of information could modulate the resulting pattern of these two analyses. Therefore, we tested whether an extended model, which considered importance as an additional variable, fitted the data better compared to the model which replicated the analysis of Kang et al. (2009). We also compared these two models with other potential models. The model comparison results revealed that curiosity was best predicted by confidence and importance (see Table 1). In line with the theoretical predictions by Dubey and Griffiths (2020), our results indicated that curiosity asymptoted on a high level for information participants were low-to-moderate confident in knowing and rated as important. The results also indicated that if information was perceived as less important, curiosity dropped and followed an inverted U-shaped function of confidence, as suggested by the information-gap theory.

Another final set of analyses sought to extend the second finding by Kang et al. (2009) and addressed whether the importance in knowing information further modulated participants’ decision to reveal an answer. As in the previous set of analyses, we compared several models with respect to BIC and found that a model which considered curiosity and importance as independent variables fitted the data best. Results of this model showed that participants were more likely to reveal the answer with increasing curiosity and also with increasing importance of knowing this information.

In sum, our results first not only replicated the results from Kang et al. (2009) but also extended these by the addition of an importance variable. We sought to test the replicability of the findings described in Experiment 1. Thus, we applied the same procedure as in Experiment 1, but with other stimulus material in another experiment.

## Experiment 2

For this new stimulus material, we chose a currently relevant subject matter that has globally impacted people in the last year and still does on a daily basis—the ongoing COVID-19 pandemic. The COVID-19 pandemic seemed like an ideal scenario to study the information-gap theory of curiosity (as intended by Kang et al., 2009), since knowing pandemic-related information dynamically changes and thus, different levels of confidence about knowing information subsist, with new regulations and vaccination policies in place on a weekly, if not, daily basis (in Germany; as of April 2021). As in Kang et al. (2009), we designed the COVID-19 questions in a way that participants should have a high variance in confidence and importance ratings on these questions.

Besides the stimulus material, the procedure for this experiment was the same as for Experiment 1. Our first intention was to replicate the findings from Kang et al. (2009) in a first set of analyses. The statistical models for these analyses were identical to the models of Experiment 1. We then sought to investigate whether these findings can be extended by an additional importance term as an independent variable. We compared the goodness of fits of several models with each other to test whether the addition of an additional importance term as an independent variable increased model fit. As in Experiment 1, we report the results of the winning model.

### Method

#### Participants

42 German participants (18 women, 22 men, *M*_age_ = 26.59 years; range 18–37) were recruited via Prolific to conduct the online study. All participants provided informed consent prior to the onset of the study. The sample size was based on the same sample size as in Experiment 1 for which power was sufficient (> 80% for a 0.05 alpha level) to reveal an interaction effect between confidence and importance on curiosity.

#### Stimuli, procedure, and data analysis

The stimuli used in Experiment 2 were 20 questions about the ongoing COVID-19 pandemic in Germany, which were adapted from information on the Robert Koch institute website (see Supplementary file for the list of COVID-19-related questions). All these questions were designed to measure curiosity about information and to evoke curiosity like the trivia questions used in Experiment 1. Example of a question about the pandemic: “Is it possible to become infected with the coronavirus after vaccination?”, Answer: “Yes” (Robert Koch Institute, 2021). The task and timeline were exactly the same as in Experiment 1. Participants rated 20 COVID-19-related questions on the three scales: curiosity, confidence, and importance. The data analysis procedure in this experiment was exactly the same as in Experiment 1. Please note that we did not pretest the 20 questions before the start of the experiment. Pretesting would have taken time and information might have changed after pretesting, due to new rules and regulations in place.

### Results

As in Experiment 1, we first removed all participants who chose to reveal all answers and no answers. The final experimental data consisted of a total of 38 participants in Experiment 2. The results are reported in Tables 5 and 6.

**Table 5:** Curiosity on COVID-19 questions predicted by confidence and importance

Predictors	Curiosity				Curiosity			
	*b*	SE	*t* value	*p*	*b*	SE	*t* value	*p*
Intercept	− 0.00	0.04	− 0.01	0.994	− 0.93	0.10	− 9.60	** < 0.001**
Confidence [1st degree]	0.34	0.97	0.35	0.723	1.03	1.77	0.58	0.559
Confidence [2nd degree]	− 3.42	0.97	− 3.53	** < 0.001**	− 4.36	1.76	− 2.48	**0.013**
Importance					0.27	0.02	12.58	** < 0.001**
Confidence [1st degree]: Importance					− 1.26	0.46	− 2.75	**0.006**
Confidence [2nd degree]: Importance					0.32	0.45	0.72	0.470
*N*	38 _participants_				38 _participants_			
AIC	2116.445				2010.199			

A smaller AIC score indicates a better model fit

Numbers are in bold for all p-values below 0.05

**Table 6:** The decision to reveal an answer (decision) on COVID-19 questions predicted by confidence and importance

Predictors	Decision				Decision			
	*b*	SE	*t* value	*p*	*b*	SE	*t* value	*p*
Intercept	− 0.57	0.19	− 2.94	**0.003**	− 1.53	0.29	− 5.22	** < 0.001**
Curiosity	0.54	0.09	6.09	** < 0.001**				
Confidence [1st degree]					1.55	5.20	0.30	0.766
Confidence [2nd degree]					− 0.79	5.30	− 0.15	0.881
Importance					0.28	0.06	4.67	** < 0.001**
Confidence [1st degree]: Importance					− 3.95	1.32	− 2.99	**0.003**
Confidence [2nd degree]: Importance					− 3.18	1.32	− 2.41	**0.016**
*N*	38 _VPcount_				38 _VPcount_			
AIC	903.772				874.416			

A smaller AIC score indicates a better model fit

Numbers are in bold for all p-values below 0.05

#### Analysis 2a: curiosity as a function of confidence

In line with the results reported by Kang et al. (2009) and the first result of Experiment 1 of an inverted U-shaped function between curiosity and confidence was replicated in this experiment (see Fig. 3). The main effect of the quadratic coefficient for confidence was significant (*b*_1_ estimate = − 3.42; *t* = − 3.53; *p* < 0.001), while the main effect for the coefficient for confidence was not (*b*_2_ estimate = 0.34; *t* = 0.35; *p* = 0.723).

#### Analysis 2b: probability of revealing the answer as a function of curiosity

As in Experiment 1, a logistic regression was then used to analyze the effect of normalized curiosity on the decision to reveal answers for the COVID-19 questions (see Fig. 4). As in Kang et al. (2009) and as in Experiment 1, the results indicated a significant effect of normalized curiosity on the probability to reveal an answer (*b* = 0.54; *z* = 6.08; *p* < 0.001). The positive relationship between curiosity and the decision to reveal the answer was also indicated by a positive and significant correlation *r* = 0.44, *p* = 0.005, and was in line with Experiment 1 and the findings by Kang et al. (2009).

#### Model comparison results

As in Experiment 1, curiosity was best explained by a model which included the 2nd order polynomial confidence term and the importance variable (see Table 1).^11^ In contrast to Experiment 1, participants’ decision to wait for an answer was best predicted by a model of confidence and importance (see Table 2). The results of these two winning models are described below as well as in Tables 5 and 6.

#### Analysis 2c: curiosity as a function of confidence and importance

This analysis revealed the same result pattern for confidence as 2a with a significant main effect of confidence on the quadratic term (*b*_1_ = − 4.386 *t* = − 2.48; *p* < 0.001), but not the linear term (*b*_2_ = 1.03; *t* = 0.58; *p* = 0.559). In addition, the main effect of importance was significant (*b*_3_ = 0.26; *t* = 12.58; *p* < 0.001). The interaction between the quadratic confidence term and importance was not significant (*b*_4_ = 0.32; *t* = 0.72; *p* = 0.47), but the interaction between the linear confidence term and importance was significant (*b*_5_ = − 1.26; *t* = − 2.74; *p* < 0.001). This interaction mimicked our predictions (see Fig. 1) that curiosity would asymptote on a high level for important and low-to-moderate confidence ratings and decrease with higher confidence ratings. Furthermore, on lower importance ratings, curiosity followed an inverted U-shaped function of confidence (see Figs. 5, S1).

#### Analysis 2d: probability of revealing the answer as a function of confidence and importance

This analysis revealed a significant main effect of confidence on the quadratic term (*b*_1_ = − 0.79; *z* = − 0.15; *p* = 0.881), and the linear term (*b*_2_ = 1.55; *z* = 0.29; *p* = 0.766). In line with our predictions on the influence of importance on curiosity, the main effect of importance was significant (*b*_3_ = 0.28; *z* = 4.66; *p* < 0.001). The interaction between the quadratic confidence term and importance was significant (*b*_4_ = − 3.18; *z* = − 2.41; *p* = 0.016), and the interaction between the linear confidence term and importance was significant (*b*_5_ = − 3.95; *z* = − 2.99; *p* = 0.003). This interaction pattern suggested that the willingness to spend time to reveal the answer—a behavioral marker for being curious—would asymptote on a high level for important and low-to-moderate confidence ratings and decrease with higher confidence ratings. Furthermore, on lower importance ratings, the decision to wait for an answer followed an inverted U-shaped function of confidence.

## Discussion

This experiment was conducted to replicate the findings from Kang et al. (2009) and the findings of Experiment 1 but with other stimulus material—namely COVID-19-related questions. Therefore, we followed the same two-step analysis procedure as reported in Experiment 1. Results replicated the findings of Kang et al. (2009) and Experiment 1 of (a) an inverted U-shaped relationship between curiosity and confidence, (b) curiosity leading to a greater willingness to wait some time to get to know the correct answer for a particular question. As in Experiment 1, the addition of the importance variable increased the model fits. Analysis 2c replicated the results of Experiment 1 showing that the importance of knowing information interacted with confidence, with low-to-moderate confidence in knowing important information leading to the highest curiosity levels. However, a comparison of model fits indicated that participants’ decision to wait for an answer was best predicted by a quadratic confidence term and the importance of knowing information. The results of this model suggested that participants were most likely to wait for an answer if this information was important and when they were not confident or moderately confident in knowing this information. We discuss these findings in the general discussion.

## General discussion

In this study, we replicated and extended previous findings investigating curiosity as a function of confidence (Dubey & Griffiths, 2020; Kang et al., 2009). In particular, we first replicated the results from Kang et al. (2009) reporting an inverted U-shaped function between curiosity and confidence (see Fig. 3) and showing that people are more inclined to spend time learning information that they are more curious about (see Fig. 4). In addition, we extended these results showing that participants’ perceived importance of information further modulated the relationship between curiosity and confidence: Participants were most curious about important information which they rated as being low-to-moderate confident in knowing (see Fig. 5). If, however, information was rated as less important, curiosity dropped overall and followed an inverted U-shaped function of confidence (see Fig. 5). These results are in line with the rational model of curiosity by Dubey and Griffiths (2020) showing that the novelty theory and the information-gap theory can be integrated within one framework by assessing the modulatory effect of the importance of knowing information.

We also found that the importance of knowing information increased participants’ likelihood of deciding to wait for an answer in addition to participants’ curiosity level. Importantly, both extended models fitted the data better compared to the model which replicated the results by Kang et al. (2009) providing evidence that the addition of the importance of knowing information contributed to the interplay of curiosity, confidence, and participants’ willingness to close information gaps. We replicated most of these findings with a second experiment applying different stimulus material regarding COVID-19-related information. Results of the final analysis of this second experiment suggested that participants’ likelihood of deciding to wait for an answer was best predicted by a model with an importance term and a quadratic term for confidence. Finally, all reported analyses considered the relationship between variables and are thus correlational. Thus, the conducted analyses do not provide evidence for a causal direction of the variables of interest measured in this study.

Importantly, the resulting pattern of both experiments suggested that people could be very curious about important information even if they were 0% confident in knowing the answer to this question. In other words, the results suggest that people are curious about information when the only thing they know about this information is that knowing the answer to it is important. This further supports the hypothesis that people can estimate the importance of information—at least for some types of information—on a metacognitive level, even if they are very unconfident in knowing the answer (Dubey & Griffiths, 2020).

This finding somewhat contradicts the hunger metaphor for curiosity, as a small bite for knowledge may not be needed to be curious about information, but rather suggests that having an estimate of the importance of information is enough to elicit curiosity. Finally, the results of the importance analyses further showed that when the importance of information declined, participants were most curious about this relatively unimportant information when they were moderately confident in knowing the information (i.e., curiosity followed an inverted U-shaped function of confidence). This indicates that curiosity may be best elicited for relatively unimportant information when participants already know a bit of this information.

So far, few other studies investigated the role of self-indicated importance of information on persons’ curiosity (Dubey et al., 2019; Golman & Loewenstein, 2018; Liquin & Lombrozo, 2020). For instance, Liquin and Lombrozo (2020) found that participants were more likely to seek information if the utility of this information was rated as high. They also found that the effect of utility on curiosity was larger than the effect of confidence on curiosity. On a similar account, Dubey et al. (2019) found that increasing the awareness of importance concerning a scientific topic also increased curiosity and that increased curiosity about a topic was followed by a higher willingness to learn about this information. Importantly, Liquin and Lombrozo (2020) as well as Dubey et al. (2019) used a different paradigm as Kang et al. (2009). In addition, these two studies investigated confidence separately from importance or utility (i.e., both as main effects) but did not investigate curiosity as a function of confidence and importance (i.e., both main effects plus the interaction between these two factors). Nevertheless, the results from Liquin and Lombrozo (2020), as well as Dubey et al. (2019), provided further evidence showing that the perceived importance of information influences participants’ curiosity.

Another question for future research concerns what people indicate as important information. For example, information gaps may be perceived as more or less important because of intrinsic motivational aspects, as some people may anticipate high learning gains, associated with rewards (Gottlieb & Oudeyer, 2018; Kang et al., 2009; Masís et al., 2021), from learning new information. Dissociating different factors contributing to the perceived importance of information may be explored by future research and could make a considerable contribution to learning about the mechanisms which elicit curiosity.

Another future avenue may consider to investigate further stimulus material to examine the generalizability of the trivia questions to other questions. For instance, we used COVID-19 questions in Experiment 2 to replicate the results of Experiment 1. The results of these questions replicated the resulting pattern of the first three analyses of Experiment 1. However, the results of the final analyses differed with importance and curiosity best-predicting participants’ decision to wait for an answer in Experiment 1 and importance and a quadratic term of confidence best-predicting participants’ decision to wait for an answer in Experiment 2. While both of these results show that adding the variable importance of information contributes to the models’ fit, they suggest that further research is needed to investigate the interplay of these variables—including further research on how participants’ decision to wait for an answer can be explained best.

In conclusion, the results of the two experiments we reported in this study support recent findings from Dubey and Griffiths (2020) suggesting that people are most curious about information gaps—whether larger or medium—which are perceived as important. However, the results also suggest that with decreasing importance, curiosity follows an inverted U-shaped function of confidence. These findings combine the information-gap theory and novelty theory within one framework and highlight that the perceived importance of knowing information plays a crucial role in our curiosity on this information.

### Supplementary Information

Below is the link to the electronic supplementary material.

Supplementary file1 (DOCX 164 kb)

## Data Availability

Raw data and commented analysis scripts are available via the Open Science Framework at https://osf.io/5tqwr/.

## Footnotes

1 As there is no general definition of curiosity, we operationalize curiosity as information-seeking—similar to other prior researchers—which can be driven by intrinsic but also extrinsic motivations (Dubey & Griffiths, 2020; Kidd & Hayden, 2015).

2 These questions were used by Kang et al. (2009) to elicit epistemic curiosity, i.e., the desire for a specific piece of information.

3 Please note that in Kang et al. (2009) participants had to wait between 5 and 25 s to reveal the answer, while the waiting time was always 10 s in Dubey and Griffiths (2020).

4 Note that Kang et al. (2009) and Dubey and Griffiths (2020) differed with respect to the independent variable when investigating the probability to reveal an answer. Kang et al. (2009) examined the effect of curiosity, while Dubey and Griffiths (2020) examined the effect of confidence and group on the probability to reveal an answer.

5 Another illustrative example supporting this claim is being asked on the confidence of knowing tomorrows winning lottery numbers. Evidently, confidence here is 0%, as these numbers do not exist yet. However, one may be able to estimate that magically dreaming the correct lottery numbers tonight could be highly important.

6 Note that we did not examine the causal relationship between curiosity and the decision to reveal an answer as participants may have made an implicit choice on whether they would like to know the answer or not before they indicated their curiosity ratings. The analysis was rather motivated to replicate the analysis reported by Kang et al. (2009).

7 Please note that this setup was similar to the first two phases of Dubey and Griffiths (2020) but with the 5–25 s waiting time (as in Kang et al., 2009) for revealing the answer to the stimulus (instead of 10 s as in Dubey & Griffiths, 2020) and without Phase 3 of Dubey and Griffiths’ study design.

8 Note that results were virtually identical when assessing non-normalized data.

9 Note that that the BIC score of another model with only curiosity as the independent variable revealed a similar BIC score (below 10 in BIC differences) but the reported model had a lower AIC.

10 Note that the relationship observed in Fig. 3 (left chart) may also indicate a potential non-monotonic relationship. The specific nature of this relationship may be addressed in future research.

11 The same model without importance had a similar BIC but the AIC differed between these two models favoring the reported model.
