# Supplementary material for: On the interplay of curiosity, confidence, and importance in knowing information
Source: Psychol Res. 2023 Jun 6;88(1):101–15. doi: 10.1007/s00426-023-01841-9 (PMC10243256; doi:10.1007/s00426-023-01841-9)
Supplement: Supplementary file 1 — Supplementary file1 (DOCX 164 kb) [file 426_2023_1841_MOESM1_ESM.docx]

**Trivia Questions Used in Experiment 1**

1. What rock and roll band performs “I wanna Rock and Roll All Night?”

Answer: KISS

1. What unfortunate handicap did Thomas Edison suffer from?

Answer: Deafness

1. What book is the most shoplifted book in the world?

Answer: The Bible

1. How long were Jerry Seinfeld and his pals sentenced in the series finale?

Answer: One year

1. What is the only type of animal besides a human that can get a sunburn?

Answer: Pig

1. What snack food is an ingredient in the explosive dynamite?

Answer: Peanuts

1. What invention should make Tsai Lun, a 2nd century inventor, a household name?

Answer: Paper

1. What breed of dog is the only animal whose evidence is admissible in American courts?

Answer: Bloodhound

1. What animal can shed up to 30,000 teeth in its lifetime?

Answer: Shark

1. Which country has the highest percentage of women in the government?

Answer: Ruanda (Original Answer: Belgium)

1. What instrument was invented to sound like a human singing?

Answer: Violin

1. What everyday food will make a drug test show up positive?

Answer: Poppy seeds

1. What industry uses 20% of harvested plants from China?

Answer: Medicine

1. What electronic item is stolen most often on the NYC subways?

Answer: iPhone

1. What is the name of the galaxy that Earth is a part of?

Answer: Milky Way

1. What is the most abundant mineral in the human body?

Answer: Calcium

1. What country has won the most Miss World beauty contests?

Answer: Venezuela

1. What is the only country in the world that has a bill of rights for cows?

Answer: India

1. What was the first animated film to win an Academy Award?

Answer: Beauty and the Beast

1. What city has the only drive-thru post office in the world?

Answer: Chicago

**COVID-19-Related Questions Used in Experiment 2**

1. Approximately what percentage of the total number of deaths due to COVID-19 are attributable to people aged over 69 years?

Answer: 89%

1. What percentage of people in Germany have already received at least one COVID-19-related vaccination as of 10 Mai?

Answer: 32.8%

1. What percentage of the population should be vaccinated in order to limit the spread of COVID-19 to such an extent that this pandemic will pass (based on the predictions of the mathematical model)?

Answer: 70%

1. How many people died in Germany as of 10 May due to COVID-19?

Answer: 84829

1. Which companies have their vaccines licensed in Germany?

Answer: vaccines of BioNtech, Pfizer, vaccines of Johnson & Johnson, vaccines of Moderna, vaccines of Oxford, AstraZeneca

1. Can pets, such as cats and dogs, become infected with the coronavirus?

Answer: Yes

1. What is the minimum distance to be kept to protect yourself and others from COVID-19 coronavirus infection?

Answer: 1.5 meters

1. Do medical masks provide sufficient protection against airborne viral infections?

Answer: No

1. How many days of quarantine is required for a person who may have been infected after contact with an infected person?

Answer: 14 days

1. Do people now wishing to enter Germany by air need to be tested for COVID-19 before departure?

Answer: Yes

1. According to current vaccination regulations, what is the recommended interval between the first and second vaccination (BioNTech, Moderna and AstraZeneca)?

Answer: 6 weeks for mRNA vaccines (BioNTech, Moderna), 12 weeks for vector-based vaccine (AstraZeneca)

1. Is it possible to become infected with the coronavirus after vaccination?

Answer: Yes

1. Do people who have been vaccinated need to continue wearing masks?

Answer: Yes

1. On average, what percentage of people without corona-related symptoms are correctly identified by rapid-antigen-test?

Answer: About 58%

1. What does the seven-day R-index mean?

Answer: Reproduction number R denotes the number of people infected by a COVID-19 infected person on average.

1. What percentage of confirmed cases of coronavirus in Germany are currently of the B.1.1.7 variant from the UK?

Answer: About 92%

1. Has the coronavirus B.1.617 variant from India been detected in Germany?

Answer: Yes

1. How many COVID-19 vaccines are now under rolling review by the European Medicines Agency (EMA)?

Answer: 4

1. For people who have been vaccinated with mRNA COVID-19 Vaccines, how much less likely are they to be infected with COVID-19 than those who have not been vaccinated?

Answer: About 95%

1. What does the seven-day incidence rate in Germany indicate?

Answer: Germany’s seven-day incidence rate shows the number of new coronavirus infections — according to positive PCT and antigen tests — within the past week per 100,000 inhabitants.

**Figure S1**


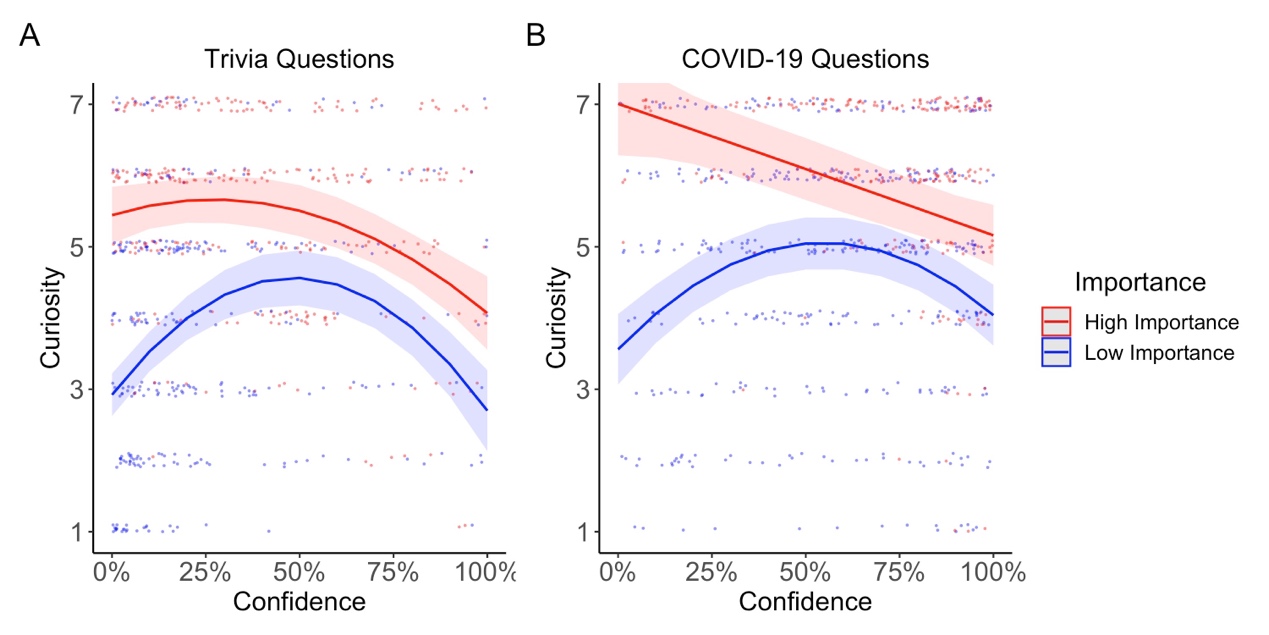


*Note.* Curiosity as a function of confidence and high/low importance ratings for trivia questions (A) and COVID-19 questions (B). Solid lines indicate the logistic regression line. The shaded area around the curves indicates the standard error of the mean. Points represent the data of individual participants jittered for each curiosity level for presentation purposes. For each experiment the median of importance was computed and data was split into two importance groups (low/high) with high values indicating importance levels above the median (Trivia importance median = 3; COVID-19 importance median = 5) and low values indicating importance levels equal or below the median.
